# Supplementary material for: Salivary Oxidative Stress Biomarkers in Peri-Implant Disease: A Systematic Review and Meta-Analysis
Source: Int J Mol Sci. 2025 Nov 21;26(23):11269. doi: 10.3390/ijms262311269 (PMC12692554; doi:10.3390/ijms262311269)
Supplement: Supplementary file 1 [file ijms-26-11269-s001.zip › Supplementary File S0. README Index.pdf]

Supplementary Materials – README Index

Title of Review: Salivary Oxidative Stress Biomarkers in Peri-Implant Disease: A Systematic Review and Meta-Analysis

Systematic Review Registration: PROSPERO ID: CRD420251117832

Statistical Software Used:
- Review Manager (RevMan) version 5.4 (The Cochrane Collaboration)
- MetaXL version 5.3 (Epigear International)

Meta-Analysis Methods:
- Effect sizes pooled as standardized mean differences (SMD) using the inverse variance method.
- Random-effects models (DerSimonian–Laird) applied for all primary analyses.
- Heterogeneity assessed via I^2 statistics.
- Subgroup analyses performed by assay type and normalization method (if applicable).
- Publication bias assessed visually via funnel plots and statistically using Egger's test where possible.

Note on Reproducibility:
All analyses were performed using the standard interface of RevMan and MetaXL. No custom scripts or user-defined macros were applied. Default program settings were used throughout. Data extraction and input were independently verified by two reviewers. All data available upon request.

List of Supplementary Files:

File	Description
S1. Full Electronic Search Strategies (.docx)	Database-specific search strings (PubMed, Scopus, Web of Science) with search dates and filters used.
S2. Risk of Bias Assessments (.docx)	Domain-level judgments using ROBINS-I and QUADAS-2 tools, including tabulated results.
S3. GRADE Evidence Profile Tables (.docx)	Certainty assessments across five domains for each biomarker (MDA, TAC), following GRADE methodology.
S4. PROSPERO Protocol	
S5. PROSPERO Deviations	
