# Supplementary material for: Salivary Oxidative Stress Biomarkers in Peri-Implant Disease: A Systematic Review and Meta-Analysis
Source: Int J Mol Sci. 2025 Nov 21;26(23):11269. doi: 10.3390/ijms262311269 (PMC12692554; doi:10.3390/ijms262311269)
Supplement: Supplementary file 1 [file ijms-26-11269-s001.zip › Supplementary File S1. Full Electronic Search Strategies.pdf]

Supplementary File S1. Full Electronic Search Strategies

This appendix details the exact database-specific search strategies used in this systematic review. All searches were conducted in September 2025 and limited to studies published in English between January 2004 and September 2025. No filters were applied for study design.

PubMed (MEDLINE)

Date of final search: September 30, 2025

Search string:

("saliva"[MeSH Terms] OR "saliva"[All Fields] OR "salivary") AND
("oxidative stress"[MeSH Terms] OR "oxidative stress"[All Fields] OR "oxidative damage" OR "redox status") AND
("biomarkers"[MeSH Terms] OR "biomarker"[All Fields] OR "marker" OR "indicator") AND
("malondialdehyde"[MeSH Terms] OR "malondialdehyde"[All Fields] OR "MDA" OR "total antioxidant capacity" OR "TAC" OR "8-hydroxydeoxyguanosine" OR "8-OHdG") AND
("peri-implantitis"[MeSH Terms] OR "peri-implantitis"[All Fields] OR "peri-implant mucositis" OR "dental implants")

Filters: English; Publication date from 2004/01/01 to 2025/09/30

Scopus

Date of final search: September 30, 2025

Search string:

(TITLE-ABS-KEY("saliva" OR "salivary") AND
TITLE-ABS-KEY("oxidative stress" OR "oxidative damage" OR "redox status") AND
TITLE-ABS-KEY("biomarker" OR "marker" OR "indicator") AND
TITLE-ABS-KEY("malondialdehyde" OR "MDA" OR "total antioxidant capacity" OR "TAC" OR "8-OHdG") AND
TITLE-ABS-KEY("peri-implantitis" OR "peri-implant mucositis" OR "dental implants"))
AND (LIMIT-TO(LANGUAGE, "English"))
AND (PUBYEAR > 2003 AND PUBYEAR < 2026)

Web of Science Core Collection

Date of final search: September 30, 2025

Search string:

TS=("saliva" OR "salivary") AND
TS=("oxidative stress" OR "oxidative damage" OR "redox status") AND
TS=("biomarker" OR "marker" OR "indicator") AND
TS=("malondialdehyde" OR "MDA" OR "total antioxidant capacity" OR "TAC" OR "8-OHdG") AND
TS=("peri-implantitis" OR "peri-implant mucositis" OR "dental implants")

Refined by: LANGUAGE: (ENGLISH)

Timespan: 2004–2025

Indexes: SCI-EXPANDED, SSCI, ESCI

Notes:

- The final search results were exported to Zotero (v6.0) for duplicate removal and manual verification.
- No automated filters for study design or article type were applied.
- Manual screening of reference lists was performed for all included articles.
- No gray literature or trial registry searches were conducted.
