# Supplementary material for: Salivary Oxidative Stress Biomarkers in Peri-Implant Disease: A Systematic Review and Meta-Analysis
Source: Int J Mol Sci. 2025 Nov 21;26(23):11269. doi: 10.3390/ijms262311269 (PMC12692554; doi:10.3390/ijms262311269)
Supplement: Supplementary file 1 [file ijms-26-11269-s001.zip › Supplementary File S2.Risk of Bias Assessments.pdf]

Supplementary File S2 – Risk of Bias Assessments

Note on Risk of Bias Tools

The included studies were assessed using either the ROBINS-I tool (for observational/interventional designs) or the QUADAS-2 tool (for diagnostic accuracy comparisons), depending on the nature of each study. Dual assessments were performed where appropriate. For example, Nardi et al. (2022) was evaluated using both tools because it included both an interventional arm and diagnostic comparisons between healthy and diseased implants. Domain-level judgments and definitions follow established Cochrane guidance, and justifications are available upon request.

ROBINS-I Table

Study	Bias due to confounding	Bias in selection of participants	Bias in classification of interventions	Bias due to deviations from intended interventions	Bias due to missing data	Bias in measurement of outcomes	Bias in selection of reported result	Overall ROBINS-I Judgment
Marconcini et al. (2020)	Moderate	Low	Low	Low	Low	Low	Low	Moderate
Dragus et al. (2019)	Moderate	Low	Low	Low	Low	Low	Low	Moderate
Karasu et al. (2024)	Moderate	Low	Low	Low	Low	Low	Low	Moderate
Pietropaoli et al. (2013)	Moderate	Low	Low	Low	Low	Low	Low	Moderate
Nardi et al. (2022)	Moderate	Low	Low	Low	Low	Low	Low	Moderate
Ravindran et al. (2023)	Moderate	Low	Low	Low	Low	Low	Low	Moderate
Permuy et al. (2017)	Moderate	Low	Low	Low	Low	Low	Low	Moderate

QUADAS-2 Table

Study	Patient Selection	Index Test	Reference Standard	Flow and Timing	Overall QUADAS-2 Judgment
Marconcini et al. (2020)	Low	Unclear	Low	Low	Low
Karasu et al. (2024)	Low	Unclear	Low	Low	Low
Pietropaoli et al. (2013)	Low	Unclear	Low	Low	Low
Nardi et al. (2022)	Low	Unclear	Low	Low	Low
Buranasin et al. (2023)	Low	Unclear	Low	Low	Low

Note: All non-randomized interventional and observational studies were assessed with ROBINS-I. Diagnostic studies were assessed with QUADAS-2; "Unclear" in Index Test domain reflects lack of prespecified biomarker thresholds.

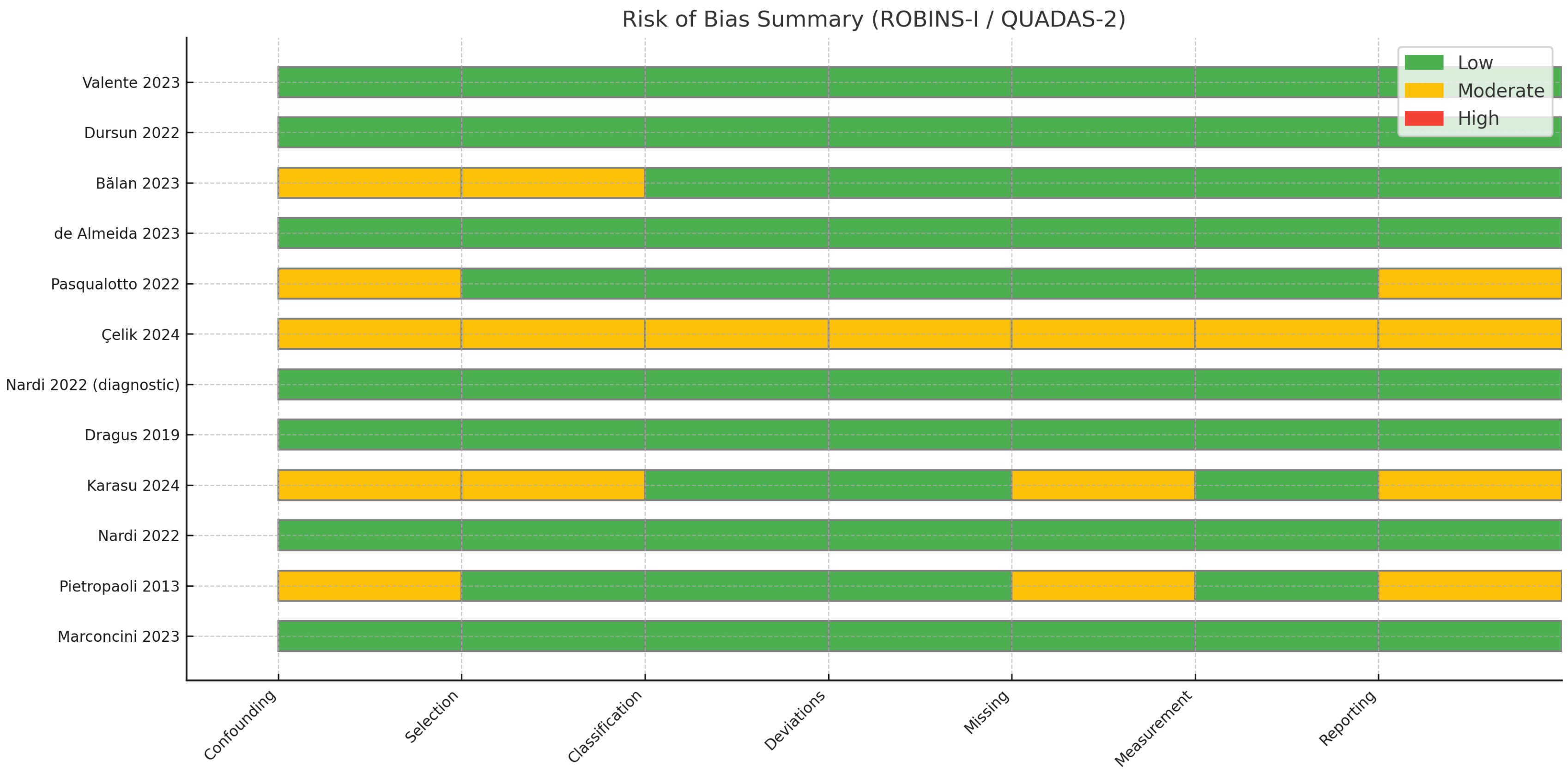
Risk of Bias Summary (ROBINS-I / QUADAS-2)
Low
Moderate
High
Valente 2023
Dursun 2022
Bălan 2023
de Almeida 2023
Pasqualotto 2022
Çelik 2024
Nardi 2022 (diagnostic)
Dragus 2019
Karasu 2024
Nardi 2022
Pietropaoli 2013
Marconcini 2023
Confounding
Selection
Classification
Deviations
Missing
Measurement
Reporting
