# Supplementary material for: Salivary Oxidative Stress Biomarkers in Peri-Implant Disease: A Systematic Review and Meta-Analysis
Source: Int J Mol Sci. 2025 Nov 21;26(23):11269. doi: 10.3390/ijms262311269 (PMC12692554; doi:10.3390/ijms262311269)
Supplement: Supplementary file 1 [file ijms-26-11269-s001.zip › Supplementary File S3. GRADE Evidence Profile Tables.pdf]

Supplementary File S3 – GRADE Evidence Profile Tables

This file contains GRADE evidence profiles for salivary oxidative stress biomarkers measured in the meta-analysis. Certainty of evidence was assessed across five domains: risk of bias, inconsistency, indirectness, imprecision, and publication bias, using GRADE guidelines. For each biomarker (MDA and TAC), explanations are provided for judgments. Pooled SMD values, heterogeneity (I^2), and visual evidence (forest plots, funnel plots) were also considered to rate the quality of evidence. Only studies included in the meta-analysis were evaluated: 7 for MDA and 5 for TAC.

GRADE Evidence Profile – MDA

GRADE Domain	Judgment	Rationale	Impact on Certainty
Risk of Bias	Serious	Two studies showed moderate risk due to unclear confounding control and selective outcome reporting (see ROBINS-I heatmap).	Downgraded one level
Inconsistency	Moderate	$I^2 \approx 0\%$; no serious inconsistency; retained conservative certainty due to observational design	Low (observational studies)
Indirectness	Not Serious	All included studies evaluated MDA in saliva of patients with confirmed peri-implant disease vs. healthy controls.	No downgrade
Imprecision	Not Serious	Some 95% CIs crossed minimal clinically important differences; sample sizes <100 per arm.	No downgrade
Publication Bias	Undetected	Funnel plot was symmetrical; Egger's test not significant with n=7 (see Funnel Plot 1).	No downgrade
Overall Certainty	Low	Evidence downgraded for risk of bias and imprecision despite consistent direction of effect.	Final GRADE: Low

GRADE Evidence Profile – TAC

GRADE Domain	Judgment	Rationale	Impact on Certainty
Risk of Bias	Some concerns	All five studies had low risk of bias; measurement tools clearly described.	No downgrade
Inconsistency	Moderate	$I^2 \approx 0\%$; no serious inconsistency; large effect size observed; however, all contributing studies were observational and at overall moderate risk of bias, so certainty was rated as Moderate.	No downgrade
Indirectness	Not Serious	TAC assessed in the correct matrix (saliva) and target population; no concerns noted.	No downgrade
Imprecision	Not Serious	Tight confidence intervals with consistent direction and clear effect separation.	No downgrade
Publication Bias	Undetected	No clear asymmetry in funnel plot; small number of studies (n=5) limits detection power.	No downgrade
Overall Certainty	Moderate	All contributing studies were observational and at overall moderate risk of bias	Final GRADE: Moderate
