# Supplementary material for: Salivary Oxidative Stress Biomarkers in Peri-Implant Disease: A Systematic Review and Meta-Analysis
Source: Int J Mol Sci. 2025 Nov 21;26(23):11269. doi: 10.3390/ijms262311269 (PMC12692554; doi:10.3390/ijms262311269)
Supplement: Supplementary file 1 [file ijms-26-11269-s001.zip › Supplementary File S4. PROSPERO Protocol.pdf]

NIHR | National Institute for Health and Care Research

PROSPERO
International prospective register of systematic reviews

Utilization of Salivary Oxidative Stress Biomarkers as Predictors of Dental Implant Success: A Systematic Review and Meta-Analysis

Paul Serban Popa, Claudia Elisabeta Popa Cazacu

Citation
Paul Serban Popa, Claudia Elisabeta Popa Cazacu. Utilization of Salivary Oxidative Stress Biomarkers as Predictors of Dental Implant Success: A Systematic Review and Meta-Analysis. PROSPERO 2025 CRD420251117832. Available from https://www.crd.york.ac.uk/PROSPERO/view/CRD420251117832.

REVIEW TITLE AND BASIC DETAILS

Review title

Utilization of Salivary Oxidative Stress Biomarkers as Predictors of Dental Implant Success: A Systematic Review and Meta-Analysis

Condition or domain being studied

Failure Of Osseointegration Of Dental Implant; Biomarker analysis; Oxidative stress; Inflammatory biomarkers; Peri-implantitis

This review focuses on adult patients receiving dental implants and investigates whether salivary oxidative stress biomarkers (e.g., 8-OHdG, MDA, TAC, SOD) are predictive of implant success, survival, or failure. It compares biomarker-based assessments with standard clinical monitoring and evaluates associations with peri-implantitis and osseointegration.

Rationale for the review

Dental implant success is traditionally evaluated through clinical and radiographic parameters; however, these methods may detect complications only after tissue damage has occurred. Oxidative stress has been implicated in peri-implant inflammation and tissue breakdown, and salivary biomarkers offer a non-invasive means of monitoring these molecular changes. By synthesizing current evidence on the predictive value of salivary oxidative stress biomarkers (e.g., 8-OHdG, MDA, TAC), this review aims to determine their potential role in early detection of implant failure or peri-implant disease. This could support more personalized and preventive strategies in implant dentistry.

Review objectives

Review Objective:

To systematically evaluate whether salivary oxidative stress biomarkers are associated with the clinical success or failure of dental implants, and to assess their predictive value for outcomes such as implant survival, osseointegration, and peri-implantitis.

Review Question:

Are salivary oxidative stress biomarkers associated with dental implant success, and can they predict outcomes such as implant survival, osseointegration, or peri-implant disease?

Keywords
Dental implants; Oxidative stress; Biomarkers; Saliva; Peri-implantitis; Implant failure; Non-invasive diagnostic methods

Country
Romania

ELIGIBILITY CRITERIA

Population

Included
Adults (≥18 years old) who have received dental implants, regardless of implant system or prosthetic rehabilitation type.

Patients with available clinical outcomes related to implant success, such as survival, osseointegration, or peri-implant health.

Studies reporting salivary oxidative stress biomarkers (e.g., 8-OHdG, MDA, TAC, SOD, glutathione, catalase) in relation to dental implants.

Excluded
Studies on pediatric populations (<18 years old).

Studies involving only animal models or in vitro conditions.

Patients with biomarker data collected exclusively from blood, tissue biopsies, or other non-salivary fluids.

Case reports, reviews, editorials, or conference abstracts without full-text data.

Intervention(s) or exposure(s)

Included
Biomarker analysis; Inflammatory biomarkers; Dental Endosseous Implant

Inclusion of studies that evaluated salivary biomarkers of oxidative stress (e.g., 8-OHdG, MDA, TAC, SOD, glutathione, catalase) in patients with dental implants. Biomarkers may have been measured at any timepoint before or after implant placement.

Excluded

Exclusion of studies that assessed oxidative stress biomarkers only in blood, tissue, crevicular fluid, or other non-salivary sources. Also excluded are studies not reporting clinical implant outcomes in relation to biomarker levels.

Comparator(s) or control(s)

Included
PICO tags selected: Usual Care; Clinical judgement

Studies using conventional clinical and radiographic evaluation of implant success (e.g., probing depth, mobility, radiographic bone loss) without incorporating salivary biomarker assessment were included as comparators when applicable.

Excluded
Studies lacking a defined comparator group or using only systemic (non-localized) measures unrelated to implant outcome were excluded from comparative analysis but may still be included narratively.

Study design

Both randomized and nonrandomized study types will be included.

Included
Randomised controlled trials (RCTs), prospective and retrospective cohort studies, case-control studies, and cross-sectional studies that evaluate salivary oxidative stress biomarkers in relation to dental implant outcomes.

Excluded
Case reports, case series with fewer than 10 patients, narrative reviews, editorials, letters to the editor, commentaries, in vitro or animal studies, and conference abstracts without full text.

Context

This review will include studies conducted in dental clinics, university-based research centers, or hospital-based oral surgery/implantology departments where patients received implant therapy. No geographic or economic restrictions will be applied. Only human clinical studies that assess salivary oxidative stress biomarkers in relation to dental implant success will be included.

TIMELINE OF THE REVIEW

Date of first submission to PROSPERO

01 August 2025

Review timeline

Start date: 1 August 2025. End date: 15 December 2025.

Date of registration in PROSPERO

01 August 2025

AVAILABILITY OF FULL PROTOCOL

Availability of full protocol

A full protocol has been written and uploaded to PROSPERO. The protocol may be accessed through this link https://www.crd.york.ac.uk/PROSPEROFILES/164325ef79c28e5721b9f2fc23d4f39d.pdf.

SEARCHING AND SCREENING

Search for unpublished studies

Both published and unpublished studies will be sought.

Main bibliographic databases that will be searched

The main databases to be searched are *Embase.com*, *MEDLINE*, *PubMed*, *SCI - Science Citation Index* and *Scopus*.

Other important or specialist databases that will be searched
Web of Science Core Collection, Cochrane Library, and Cochrane CENTRAL

Search language restrictions

The review will only include studies published in English.

Search date restrictions

Databases will be searched for articles published from 1 January 2009 and before by 31 July 2025.

Other methods of identifying studies

Other studies will be identified by: *looking through all the articles that cite the papers included in the review ("snowballing")*, *reference list checking*, *searching conference proceedings* and *searching trial or study registers*.

Additional information about identifying studies
Manual searching of relevant implantology and periodontology journals. Grey literature searches via Google Scholar and clinicaltrials.gov will also be performed to identify unpublished or ongoing studies.

Link to search strategy

A full search strategy is available in the full protocol as described in the *Availability of full protocol* section

Selection process

Studies will be screened independently by at least two people (or person/machine combination) with a process to resolve differences.

Other relevant information about searching and screening

Title and abstract screening, as well as full-text eligibility assessment, will be conducted independently by two reviewers. Disagreements will be resolved through discussion or consultation with a third reviewer. Duplicates will be removed using reference management software (e.g., EndNote or Rayyan). Snowballing techniques and reference list checking will supplement database searches to ensure completeness.

DATA COLLECTION PROCESS

Data extraction from published articles and reports

Data will be extracted independently by at least two people (or person/machine combination) with a process to resolve differences.

Authors will be asked to provide any required data not available in published reports.

Study risk of bias or quality assessment

Risk of bias will be assessed using: *Cochrane RoB-2* and *Newcastle-Ottawa*

Data will be assessed independently by at least two people (or person/machine combination) with a process to resolve differences.

Additional information will be sought from study investigators if required information is unclear or unavailable in the study publications/reports.

Reporting bias assessment

Funnel plots and Egger's test will be used to assess publication bias if sufficient studies are available. Selective reporting will be considered during full-text review. Sensitivity analyses will be conducted if missing results are suspected to influence the findings.

Certainty assessment

The certainty of evidence will be assessed using the GRADE approach across key outcomes, considering factors such as risk of bias, inconsistency, indirectness, imprecision, and publication bias. Each outcome will be rated as high, moderate, low, or very low certainty. Summary of Findings tables will be created where applicable.

OUTCOMES TO BE ANALYSED

Main outcomes

Primary outcomes include dental implant success and survival rates, osseointegration, and occurrence of peri-implantitis. Data will be extracted at the longest available follow-up. Acceptable measures include clinical stability, radiographic bone level, and absence of inflammation. Effect measures: odds ratio (OR), risk ratio (RR), and standardized mean difference (SMD).

Additional outcomes

Levels of salivary oxidative stress biomarkers (e.g., 8-OHdG, MDA, TAC, SOD, glutathione, catalase) will be extracted at baseline and/or post-implant placement. Acceptable measurement methods include ELISA, spectrophotometry, and HPLC. Effect measures will include standardized mean differences (SMD) or mean differences (MD) between implant success and failure groups.

PLANNED DATA SYNTHESIS

Strategy for data synthesis

Data will be synthesized quantitatively using meta-analysis when studies are sufficiently homogeneous in terms of population, interventions, and outcomes. Random-effects models will be used due to expected clinical and methodological variability. Effect sizes will include odds ratios (OR), risk ratios (RR), and standardized mean differences (SMD), with 95% confidence intervals. Statistical heterogeneity will be assessed using the I^2 statistic and Chi^2 test. Where meta-analysis is not appropriate, a narrative synthesis will be presented.

CURRENT REVIEW STAGE

Stage of the review at this submission

Review stage	Started	Completed
Pilot work	✓	
Formal searching/study identification		
Screening search results against inclusion criteria		
Data extraction or receipt of IPD		
Risk of bias/quality assessment		
Data synthesis		

Review status
The review is currently planned or ongoing.

Publication of review results
Results of the review will be published in English.

REVIEW AFFILIATION, FUNDING AND PEER REVIEW

Review team members
Assistant Professor Paul Serban Popa (review guarantor and contact) ORCID: 0000-0003-1096-9280. Universitatea "Dunărea de Jos" din Galați. Romania.

No conflict of interest declared.

Ms Claudia Elisabeta Popa Cazacu. Universitatea "Dunărea de Jos" din Galați. Romania.

No conflict of interest declared.

Named contact
Assistant Professor Paul Serban Popa (paul.popa@ugal.ro). ORCID: 0000-0003-1096-9280. Universitatea "Dunărea de Jos" din Galați. Romania.

Review affiliation
Universitatea "Dunărea de Jos" din Galați

Funding source
Review has no specific/external funding but is supported by guarantor/review team (non-commercial) institutions.

Additional information about funding

This review is supported by the Department of Dental Medicine, Faculty of Dental Medicine, "Dunărea de Jos" University of Galați, Romania.

Peer review

There has been no peer review of this planned review.

ADDITIONAL INFORMATION

Additional information

This review is part of a doctoral research project focused on salivary biomarkers and oral implantology. It aims to clarify the predictive role of oxidative stress in implant outcomes and contribute to the development of non-invasive diagnostic tools for clinical monitoring in implant dentistry.

Review conflict of interest

Declared individual interests are recorded under team member details.. No additional interests are recorded for this review.

Medical Subject Headings

Dental Implants; Osseointegration; Peri-Implantitis; Biomarkers; Saliva; Oxidative Stress; 8-Hydroxy-2'-Deoxyguanosine; Malondialdehyde; Glutathione; Superoxide Dismutase; Catalase; Meta-Analysis as Topic; Predictive Value of Tests

SIMILAR REVIEWS

Check for similar records already in PROSPERO

PROSPERO identified a number of existing PROSPERO records that were similar to this one (last check made on 1 August 2025). These are shown below along with the reasons given by that the review team for the reviews being different and/or proceeding.

- The Impact of Diabetes Mellitus on the Success, Osseointegration, and Peri-Implant Health of Dental Implants: A Systematic Literature Review [published 7 April 2025] [CRD420251007729]. The review was judged **not to be similar**
- Comparative evaluation of type 2 diabetic and non diabetic patients with dental implants for risk peri-implant mucositis , peri-impantitis and failure of osseointegration. [published 1 February 2025] [CRD42025640138]. The review was judged **not to be similar**
- "Comparison of Implant Survival and Peri-Implant Health in Prediabetic and Systemically Healthy Patients with Adjacent Implants: A 5-Year Follow-Up Systematic Review" [published 19 May 2025] [CRD420251052690]. The review was judged **not to be similar**

PROSPERO version history

- Version 1.0, published 01 Aug 2025

Disclaimer

The content of this record displays the information provided by the review team. PROSPERO does not peer review registration records or endorse their content.

PROSPERO accepts and posts the information provided in good faith; responsibility for record content rests with the review team. The guarantor for this record has affirmed that the information provided is truthful and that they understand that deliberate provision of inaccurate information may be construed as scientific misconduct.

PROSPERO does not accept any liability for the content provided in this record or for its use. Readers use the information provided in this record at their own risk.

Any enquiries about the record should be referred to the named review contact
