# Supplementary material for: Salivary Oxidative Stress Biomarkers in Peri-Implant Disease: A Systematic Review and Meta-Analysis
Source: Int J Mol Sci. 2025 Nov 21;26(23):11269. doi: 10.3390/ijms262311269 (PMC12692554; doi:10.3390/ijms262311269)
Supplement: Supplementary file 1 [file ijms-26-11269-s001.zip › Supplementary File S5. PROSPERO Deviations.pdf]

Category	Planned in PROSPERO?	Final Implementation	Notes/Justification
Outcome prioritization	Yes	Yes	Primary outcomes (MDA, TAC) unchanged
Inclusion of 8-OHdG	No	Qualitative only	Excluded from meta-analysis due to data format heterogeneity
Subgroup analyses (assay type)	Not specified	Performed post-hoc	Added based on observed variability during data extraction
Language filter	Yes (English only)	Yes	No change
Search date extension	Yes (planned Sept 2025)	Completed as scheduled	No update needed

Outcome scope adjustment: The original protocol specified analysis of the association between salivary oxidative stress biomarkers and dental implant survival/success, with potential predictive implications. No eligible longitudinal studies reporting implant failure, disease progression, or predictive performance were identified. Consequently, the review scope was narrowed to cross-sectional associations between biomarker levels and the presence of peri-implant disease (mucositis/implantitis) versus healthy controls. This adjustment is a deviation from the registered protocol and has been transparently reported in the Methods and Discussion.

Pooling of disease categories: The protocol allowed for analysis of peri-implantitis and peri-implant mucositis as separate diseased groups. Due to the small number of eligible studies for each condition, and because several included studies reported mixed groups or did not separate conditions, peri-implant mucositis and peri-implantitis were pooled into a single "peri-implant disease" category for meta-analysis. This decision was made post hoc to preserve statistical power and is acknowledged in the Discussion as a potential source of clinical heterogeneity.

Databases deviation: *The original protocol prespecified searching PubMed/MEDLINE, Embase, Web of Science Core Collection, Scopus, and CENTRAL. In practice, Embase and CENTRAL were not searched directly. This decision was based on resource constraints and anticipated high duplication with Scopus (which indexes most Embase coverage) and with PubMed for MEDLINE records. A preliminary test search indicated no unique eligible records from Embase or CENTRAL beyond those identified in Scopus, Web of Science, and PubMed. The omission is acknowledged as a deviation and may have marginally reduced retrieval of non-indexed or very recent records.*

Grey literature/registries deviation: *The protocol also prespecified searches of grey literature sources and clinical trial registries (e.g., OpenGrey, ClinicalTrials.gov, WHO ICTRP). These searches were not performed due to the focus of the review on salivary biomarker quantification, which is typically reported in peer-reviewed journals, and because preliminary*

scoping identified no ongoing or unpublished trials meeting the inclusion criteria. This omission is acknowledged as a deviation and may have excluded a small number of unpublished or non-indexed studies; however, the impact on the overall conclusions is expected to be minimal given the nature of the evidence base.
